# Patient symptoms, confidence, and adherence during the first 8 weeks of targeted oral anticancer agent treatment

**DOI:** 10.1007/s00520-026-10421-7

**Published:** 2026-02-13

**Authors:** Amna Rizvi-Toner, Antoinette B. Coe, Christopher R. Friese, Milisa Manojlovich, Lauren P. Wallner, Karen B. Farris

**Affiliations:** 1 Department of Clinical Pharmacy, University of Michigan College of Pharmacy, Ann Arbor, MI USA; 2 Center for Improving Patient and Population Health and Rogel Cancer Center, University of Michigan, Ann Arbor, MI USA; 3 School of Nursing and Rogel Cancer Center, University of Michigan, Ann Arbor, MI USA; 4 School of Nursing, University of Michigan, Ann Arbor, MI USA; 5 Departments of Internal Medicine and Epidemiology, Rogel Cancer Center, University of Michigan, Ann Arbor, MI USA

**Keywords:** Oral anticancer agents, Side effects, Symptom management, Confidence, Medication adherence

## Abstract

**Purpose:**

We aimed to understand patients’ initial experiences with targeted oral anticancer agents (OAAs). We investigated symptoms experienced and how symptom severity affected patient confidence to manage and seek care for symptoms and OAA adherence.

**Methods:**

We conducted a longitudinal prospective cohort study of patients during the first 8 weeks of targeted OAA treatment at an NCI-designated cancer center. Participants completed patient-reported outcome measures (PROMs) online at three timepoints. Descriptive statistics quantified demographics, cancer characteristics, symptom severity, confidence, and OAA adherence. Logistic regression was used to estimate confidence and adherence by each symptom at each timepoint. Mixed effects logistic regressions accounted for repeated measures and time effects on outcomes.

**Results:**

Participants (*n* = 59) reported severe symptoms at all timepoints. Tiredness and drowsiness were most frequently reported as severe. Participants’ confidence increased from timepoint 1 to 3. Most participants reported high confidence (61–86%) and excellent adherence (75–80%) across all timepoints, but 20–25% had less than excellent OAA adherence. High confidence to manage symptoms was positively associated with older age. Confidence to manage symptoms was inversely related to the severity of depression, tiredness, drowsiness, constipation, and tingling/numbness.

**Conclusion:**

Confidence to manage symptoms increased with time on OAAs, but severe symptoms persisted. Although self-reported OAA adherence was high, a notable number of participants reported suboptimal adherence. Relationships between confidence, symptom severity, and adherence should be identified in clinical settings to evaluate patients who may need extra clinical support during OAA treatment.

## Introduction

Cancer care delivery now involves greater use of oral anticancer agents (OAAs) [1–3]. Patients self-administer OAAs, their cancer treatment, typically reducing the number of required in-person clinic visits [3]. Decreased contact between patients and clinicians, while convenient for both, raises concerns about unnoticed and unaddressed symptomatic side effects and suboptimal adherence [4, 5]. Patients’ self-monitoring burden increases, requiring recognition of symptomatic side effects and severity assessment for appropriate response, either via self-management or unplanned healthcare services [6]. Successful patient symptom self-management can help maintain quality of life, prevent premature treatment discontinuation, and avoid unnecessary and costly healthcare services, e.g., emergency department (ED) visits and hospitalizations [4, 6–8].

Targeted OAAs, initially believed to have fewer and milder toxicities thus fewer symptoms, are causing patients to experience skin, cardiovascular, and gastrointestinal symptoms that dramatically impede quality of life and require attentive monitoring [2, 3]. Side effects are the main reason for patients’ targeted OAA nonadherence, which can lead to treatment failure [4, 5]. Providing patients with the necessary support to self-manage OAA symptoms will ensure sustained effective treatment, while minimizing the humanistic and financial burdens associated with unplanned healthcare use [7, 9]. Self-management is increasingly emphasized to mitigate symptoms, but many patients lack adequate education and support and report being least prepared to manage their most concerning symptoms [6, 9, 10].

To self-manage OAAs successfully, patients must be knowledgeable, confident, and self-efficacious [6, 9–13]. Self-efficacy is one’s belief in their ability to implement behaviors to achieve an outcome, while confidence is one’s belief in the ability to perform a behavior [11, 12]. Self-efficacy is commonly measured by asking patients if they were confident in performing a behavior, and in this paper, self-efficacy and confidence are used interchangeably to refer to patients’ belief in their ability to manage symptoms [11, 12]. Limited literature investigates confidence in patients taking OAAs, but studies have found that those with more confidence to self-manage fatigue and nausea had greater patient activation [13]. A crucial component of sufficient self-management, patient activation encompasses motivation, knowledge, skills, and confidence to make decisions regarding one’s health [13–15]. Chronically ill patients had fewer hospitalizations and ED visits, alongside better quality of life, with greater confidence [14, 15]. Additionally, patients with higher, versus lower, self-efficacy/confidence had higher OAA self-management scores [16]. We do not know how confident patients approach seeking care for their symptoms, which presumably impacts their reliance on self-management. Critically, few studies have evaluated symptom management in patients taking OAAs, the self-efficacy/confidence to do so, and any impacts on OAA adherence.

Medication adherence behavior is important to understand alongside confidence to self-manage OAA symptoms. A wide range of OAA adherence rates has been reported, from lower than 20% to 100%, and it often decreases over time [1]. Further exploring the relationship between OAA adherence and symptom severity will allow us to better support patients. Side effects are among the most common reasons for OAA nonadherence [4, 5, 17–19]. OAA adherence is also influenced by insurance status, informal and formal support, cost, and patient age [20–23]. Symptom relief and preventing nonadherence are likely possible with comprehensive patient monitoring and early clinician intervention. Monitoring and intervening successfully require a better understanding of factors impacting patients’ ability to self-manage, like confidence and symptom severity, and their influence on OAA adherence.

In summary, we do not fully understand patients’ experiences and behaviors while taking OAAs at home. Learning more about patients’ confidence to manage their symptoms and/or seek care in addition to their symptom severity and OAA adherence, and how/if these factors change over time, will help us provide better support. Our exploratory study’s objective was to understand patients’ experiences during the first 8 weeks of OAA therapy, including their symptoms, symptom severity, confidence to manage symptoms and seek care for them, and OAA adherence.

## Methods

### Study model

**Fig. 1:**
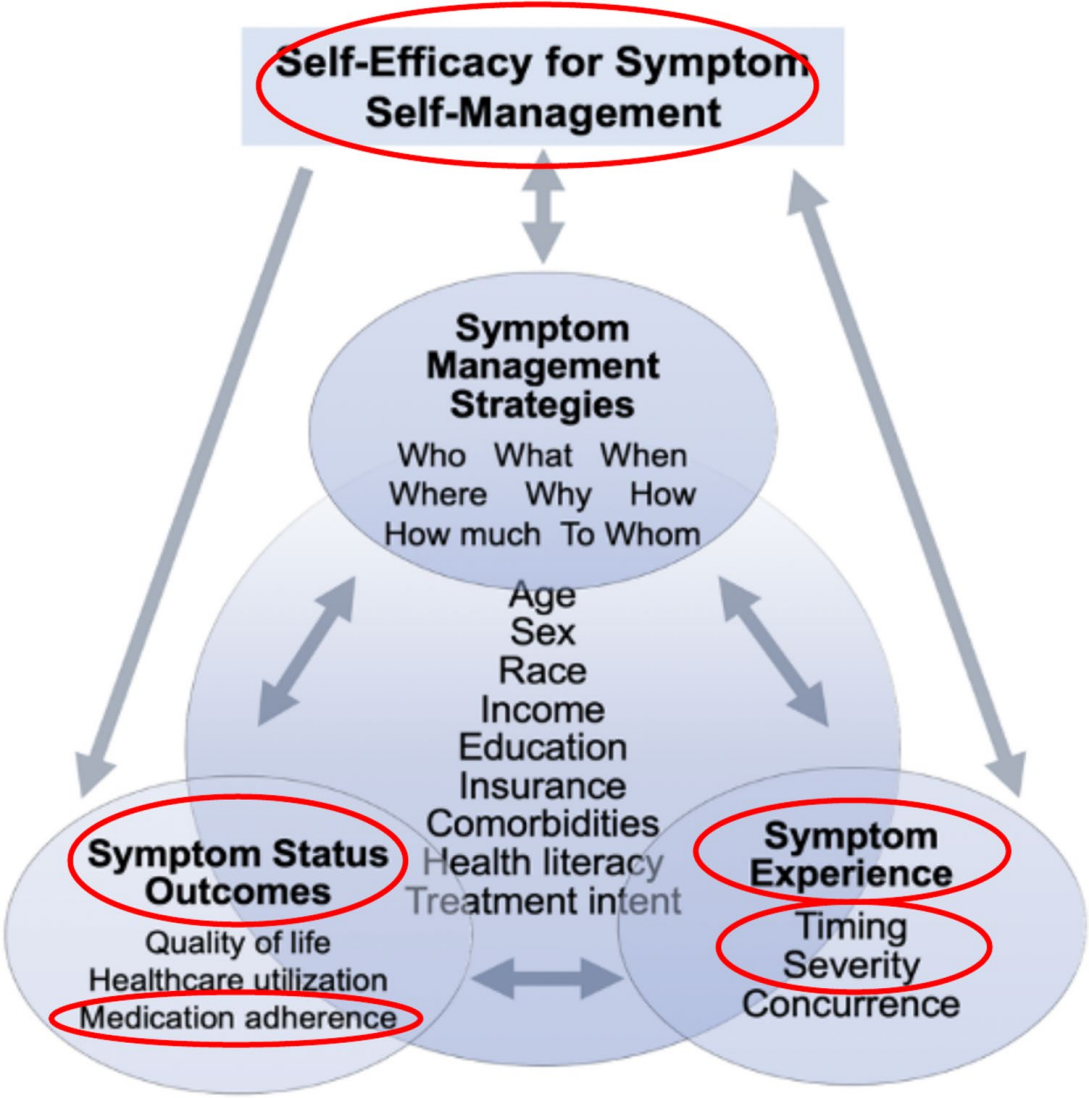
Adapted conceptual model of symptom self-management; circled components indicate the variables explored further in this study: symptom severity and timing, confidence, and adherence

The overall study was guided by an adapted conceptual model of symptom self-management and informed these analyses evaluating symptom severity, confidence, and adherence (Fig. 1). Detailed methods and overall study protocol have been published [24]. Briefly, the conceptual model (Fig. 1) posits that symptom experience, outcomes, and management strategies influence each other, while emphasizing that confidence or self-efficacy for symptom self-management affects patients’ ability to carry out symptom management behaviors. Demographics and treatment characteristics (center variables) can influence all three and, ideally, serve as controls [25, 26].

### Study design

This prospective cohort study analyzes quantitative data from a parallel convergent mixed methods study [24]. Participants completed identical patient-reported outcome measures (PROMs) within 3 weeks of targeted OAA initiation, and then every 2 weeks thereafter, to capture a total of three timepoints and a duration of approximately the first 8 weeks of treatment. The Institutional Review Board of the University of Michigan and the Rogel Cancer Center Protocol Review Committee granted study approval.

### Participants

Adult patients starting a targeted OAA as a part of their cancer treatment were recruited from an academic/NCI-designated comprehensive cancer center. Patients with an electronic health record (EHR) documented cognitive impairment were excluded. Eligible patients were identified via EHR and emailed a participation invite. Interested patients could email or call to learn more and, if wanting to participate, were enrolled after providing written informed consent. Recruitment/enrollment occurred October 2022-August 2024.

### Data collection

Data were collected using Qualtrics. Participants were emailed Qualtrics links at each timepoint to complete PROMs. The PROMs relevant to these analyses were the Edmonton Symptom Assessment System Revised (ESAS-r), confidence to self-manage symptoms, confidence to seek care for symptoms, and OAA adherence. These PROMs consisted of 19 items and were completed at each timepoint. PROMs/items, as viewed by participants online in Qualtrics, are provided in supplementary materials (Supplementary Information S.I. Figure 1). Participant demographics and clinical characteristics were obtained via EHR using a structured tool.

#### Symptom severity

Participants rated their symptom severity using the modified ESAS-r [27]. Thirteen symptoms were each rated from 0 (absence of symptom) to 10 (worst possible severity of symptom). Symptoms were categorized as mild (1–3), moderate (4–6), or severe (7–10). Participants also rated their “Best Well Being” from 0 (worst possible) to 10 (best possible) and listed their most bothersome symptom at each timepoint as a short answer response.

#### Confidence

Two questions, adapted from the patient activation measure and used in previous work, assessed confidence [13]. The first asked about confidence to manage symptoms. The second asked about confidence to know when to seek care for symptoms. A scale of 0 (not confident) to 10 (most/highest confidence) was used for both questions. Confidence levels were categorized as less than high (0–6) and high (7–10).

#### Adherence

Medication adherence was assessed via two items. The first was developed from an HIV medication single-item self-rating adherence scale [13, 28]. Patients rated their ability to take their OAA as prescribed over the past 4 weeks as excellent, very good, good, fair, or poor. Excellent adherence was compared to all other responses, which were classified as less than excellent adherence/nonadherence/suboptimal. Participants not selecting “Excellent” then marked all applicable nonadherence reasons from a list of 12. (S.I. Figure 1).

### Analysis

Descriptive statistics quantified participants’ demographics, clinical characteristics, symptom severity, confidence, and OAA adherence. Individual logistic regressions estimated both confidence and adherence by each symptom at each timepoint. To understand the effects of time and repeated measures per participant, mixed effects logistic regressions estimated participant confidence to manage symptoms by symptom severity. All analyses were conducted using R, version 4.4.2.

## Results

**Fig. 2:**
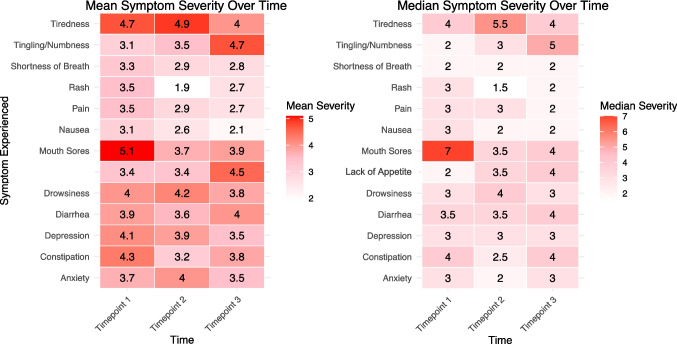
Heatmaps displaying mean and median symptom severity over time in participants who reported experiencing the symptom. Participants who did not report any level of severity (0), meaning the symptom was not experienced, were excluded

Sixty-two participants signed consent, 59 completed PROMs at timepoint 1 and 50 remained active at timepoint 3. (S.I. Figure 2 CONSORT flow diagram) Most participants were female (61%) and White (90%) with a median age of 60 years at enrollment (Table 1). More than a third of participants were diagnosed with metastatic cancer (44%). The top three cancer sites were breast (*n* = 13), hematologic (*n* = 10), and kidney (*n* = 8). The top three targeted OAAs taken were abemaciclib (*n* = 8), cabozantinib (*n* = 6), and palbociclib (*n* = 5) (Table 1).

**Table 1:** Demographics and cancer characteristics of study population (*N* = 59)

Categories	No. (%)
Age, mean (SD); range	58 (15.3); 20–84 years
Age, median (IQR)	60 (23.5)
Age groups	
18–29 years	3 (5)
30–39 years	4 (7)
40–49 years	11 (19)
50–59 years	11 (19)
60–69 years	15 (25)
70–79 years	13 (22)
80–89 years	2 (3)
Sex	
Female	36 (61)
Male	23 (39)
Race	
White	53 (89)
Black	1 (2)
Asian	3 (5)
Other^a^	2 (4)
Top 5 primary cancer locations	
Breast	13 (22)
Hematologic	10 (17)
Kidney	8 (14)
Gastrointestinal	7 (12)
Lung	5 (8)
Thyroid	4 (7)
Metastatic cancer	26 (44)
Prescribed OAA—generic name	
Abemaciclib	8 (14)
Cabozantinib	6 (10)
Palbociclib	5 (8)
Axitinib	4 (7)
Imatinib	4 (7)
Ivosidenib	3 (5)
Zanubrutinib	3 (5)
Acalabrutinib, Lapatinib, Lorlatinib, Osimertinib, Pazopanib	2 (3) each
Alectinib, Alpelisib, Asciminib, Avapritinib, Capivasertib, Dabrafenib and trametinib, Encorafenib and binimetinib, Lenvatinib, Momelotinib, Olaparib, Pacritinib, Pexidartinib, Selpercatinib, Sotorasib, Sunitinib, Venetoclax	1 (2) each

^a^Other includes those who selected other (*n* = 1) and undisclosed (*n* = 1)

Abbreviations: *SD* standard deviation, *IQR* interquartile range

Participants (*n* = 8) lost at timepoint 2 were demographically comparable to participants who remained. Four reported at least one severe symptom, and three reported less than excellent adherence (2/3 had at least one severe symptom; 1/3 had less than high confidence). Two participants completed PROMs at timepoint 1 and 3, but not timepoint 2. Three participants completed PROMs at timepoint 1 and 2, but not timepoint 3, of which two reported less than high confidence to manage symptoms, less than excellent adherence, and at least one severe symptom at timepoint 2.

### Descriptive analyses

**Fig. 3:**
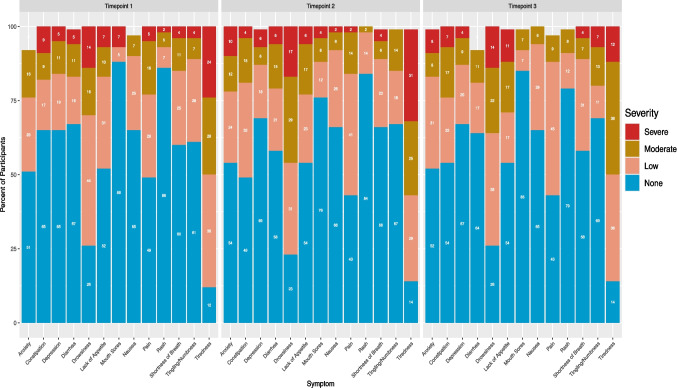
Percent of participants reporting each symptom as severe (> 7), moderate (4–6), or low (1–3) at each timepoint. Participants who selected 0 (“None”) did not have the symptom. Total percent possible for each symptom is 100; some symptoms total to less than 100 due to missingness (participant(s) did not answer)

At timepoint 1, there were 56 total instances of severe symptoms followed by 46 at timepoint 2, and 39 at timepoint 3 with 22, 21, and 16 unique participants reporting at least one severe symptom, respectively. Two participants at timepoints 1 and 3 reported no symptoms, and only one reported no symptoms at timepoint 2. Figure 2 showcases mean and median symptom severities across time. Tiredness and drowsiness were most frequently ranked as severe (≥ 7) at all timepoints (Fig. 3). Severe symptoms were reported at all timepoints, but participants reporting severe tiredness decreased from timepoint 1, 24% (*n* = 14), to timepoint 3, 12% (*n* = 6). The proportion of participants with severe drowsiness remained at 14% from timepoint 1 (*n* = 8) to timepoint 3 (*n* = 7). Figure 3 visualizes distributions of participants’ symptom severities across time. The most frequently reported most bothersome symptoms in short answer form throughout were fatigue/tiredness (*n* = 14, *n* = 9, *n* = 6) and diarrhea (*n* = 6, *n* = 12, *n* = 5), respectively.

Tiredness was the most frequently reported symptom at all timepoints, followed by drowsiness and pain (Fig. 3). The fourth most common symptom was anxiety at timepoints 1 and 3, and constipation at timepoint 2. Approximately one-third of participants reported constipation and lack of appetite at timepoints 2 and 3. Mouth sores, constipation, and diarrhea occurred more at timepoint 2 vs. 1, but then returned closer to timepoint 1 levels by timepoint 3. Shortness of breath was the only symptom experienced less at timepoint 2, 66% had none, than at timepoint 1 (60%), but then at timepoint 3 increased back near timepoint 1 levels (58%).

Confidence to manage symptoms and confidence to seek care for symptoms were the lowest at timepoint 1 with means/medians of 7/8 and 8/9, respectively. While participants generally reported high confidence (≥ 7), confidence to manage symptoms was lower than confidence to seek care overall. Less than high (≤ 6) confidence to manage symptoms was reported by 39% (*n* = 23), 14% (*n* = 7), and 26% (*n* = 11) at timepoints 1, 2, and 3, respectively. Comparably, confidence to seek care was reported less than high (≤ 6) by 15% (*n* = 8), 8% (*n* = 4), and 14% (*n* = 5) at timepoints 1, 2, and 3, respectively (S.I. Tables 1 and 2).

**Table 2:** Unadjusted logistic regressions using symptom severity, key demographics, and OAA adherence separately to estimate high confidence to self-manage OAA symptoms (≥ 7 vs. 0–6) at each study timepoint

	**Timepoint 1**		**Timepoint 2**			**Timepoint 3**	
Predictor	OR	95% CI	OR	95% CI	OR		95% CI
Pain	0.99	0.78–1.28	0.69	0.44–1.03	1.01		0.72–1.49
Tiredness	0.82	0.66–1.00	**0.69**	**0.45–0.93**	0.94		0.72–1.24
Drowsiness	**0.80**	**0.65–0.96**	0.84	0.61–1.14	0.94		0.73–1.22
Nausea	1.17	0.87–1.67	0.72	0.45–1.15	0.76		0.45–1.30
Loss of appetite	1.06	0.84–1.37	0.72	0.49–1.03	0.97		0.77–1.26
Shortness of breath	1.19	0.91–1.69	0.75	0.50–1.15	0.88		0.64–1.23
Depression	0.80	0.61–1.00	0.80	0.60–1.07	0.84		0.61–1.17
Anxiety	0.91	0.73–1.13	**0.78**	**0.60–0.995**	0.99		0.75–1.35
Constipation	0.90	0.72–1.11	0.72	0.49–1.01	**0.76**		**0.56–0.999**
Diarrhea	1.07	0.85–1.42	1.14	0.78–2.09	0.89		0.70–1.15
Tingling/numbness	**0.73**	**0.52–0.98**	0.82	0.57–1.22	0.84		0.64–1.10
Mouth sores^a^	0.91	0.69–1.19	0.77	0.55–1.10	NA		NA
Rash	0.73	0.43–1.07	1.02	0.47–4.59	1.32		0.76–3.87
Age	**1.04**	**1.00–1.08**	**1.08**	**1.02–1.17**	1.01		0.97–1.05
Male	1.83	0.62–5.76	1.9	0.36–14.28	0.73		0.19–2.96
Excellent OAA adherence	1.30	0.37–4.47	3.38	0.57–18.51	**5.33**		**1.17–25.64**

^a^Mouth sores excluded from timepoint 3 model due to low reported incidence

Most participants (75–80%) reported excellent adherence at each time point. Suboptimal adherence, defined as any value other than excellent, was reported by 25% (*n* = 14) at time point 1, 22% (*n* = 11) at time point 2, and 20% (*n* = 8) at time point 3 (See S.I. Table 3 for detailed breakdown).

**Table 3:** Fifteen separate logistic mixed effects models using symptom severity, age, OAA adherence and timepoint to estimate high confidence to manage OAA symptoms (≥ 7 vs 0–6). *Statistically significant predictors. Null model Intraclass Correlation Coefficient (ICC): 0.186

Model	ICC	Predictor	OR	95% CI
1	0.157	Depression*	0.80	0.65–0.97
		Timepoint 2*	4.38	1.58–14.22
		Timepoint 3	2.27	0.88–6.30
2	0.206	Tiredness*	0.81	0.66–0.95
		Timepoint 2*	5.43	1.88–19.29
		Timepoint 3	1.96	0.76–5.35
3	0.246	Drowsiness*	0.82	0.67–0.97
		Timepoint 2*	6.00	1.97–23.08
		Timepoint 3	2.20	0.84–6.21
4	0.136	Constipation*	0.81	0.66–0.96
		Timepoint 2*	5.31	1.85–18.49
		Timepoint 3	2.34	0.92–6.52
5	0.17	Tingling/numbness*	0.77	0.60–0.94
		Timepoint 2*	5.73	1.97–20.79
		Timepoint 3*	2.68	1.03–7.70
6	0.158	Age*	1.04	1.01–1.08
		Timepoint 2*	5.10	1.83–17.22
		Timepoint 3	2.45	0.97–6.63
7	0.209	Excellent adherence*	2.67	0.99–7.63
		Timepoint 2*	4.81	1.70–16.40
		Timepoint 3	2.21	0.86–6.15
8	0.276	Pain	0.93	0.73–1.18
		Timepoint 2*	5.01	1.74–17.56
		Timepoint 3	2.13	0.82–5.93
9	0.185	Nausea	0.96	0.74–1.28
		Timepoint 2*	5.06	1.78–17.52
		Timepoint 3	2.37	0.93–6.54
10	0.173	Appetite	0.95	0.79–1.15
		Timepoint 2*	5.00	1.78–17.06
		Timepoint 3	2.25	0.89–6.20
11	0.21	Shortness of breath	0.99	0.79-inf
		Timepoint 2*	5.86	1.92–22.93
		Timepoint 3	2.17	0.83–6.14
12	0.233	Anxiety	0.87	0.72–1.05
		Timepoint 2*	4.66	1.64–16.01
		Timepoint 3	2.07	0.81–5.66
13	0.228	Diarrhea	1.01	0.00–1.26
		Timepoint 2*	4.76	1.64–16.63
		Timepoint 3	1.97	0.77–5.42
14	0.19	Mouth sores	0.92	0.73–1.17
		Timepoint 2*	5.35	1.88–18.50
		Timepoint 3	2.34	0.92–6.41
15	0.265	Rash	0.95	0.68-inf
		Timepoint 2*	4.91	1.71–17.33
		Timepoint 3	2.26	0.88–6.22

### Estimating confidence and adherence by symptom severity at each timepoint

High confidence to manage OAA symptoms was estimated by symptom severity in separate logistic regressions for each symptom, at all timepoints (Table 2). Symptoms that were statistically significant in estimating confidence to manage OAA symptoms varied by timepoint (Table 2). At timepoint 1, participants with more severe drowsiness and tingling/numbness had lower odds of reporting high confidence than those with less severe drowsiness (OR = 0.80; 95% CI, 0.65–0.96) and tingling/numbness (OR = 0.73; 95% CI, 0.52–0.98). Also, at timepoint 1, older participants had higher odds of reporting high confidence than younger participants (OR = 1.04; 95% CI, 1.00–1.08). Older participants continued to have higher odds of reporting high confidence than younger participants at timepoint 2 (OR = 1.08; 95% CI, 1.02–1.17). Additionally, at timepoint 2, participants with greater severity of tiredness and anxiety had lower odds of reporting high confidence than those with less severe tiredness (OR = 0.69; 95% CI, 0.45–0.93) and anxiety (OR = 0.78; 95% CI, 0.60–0.995). At timepoint 3, participants with greater constipation severity had a 24% lower odds of reporting high confidence to manage OAA symptoms (OR 0.76; 95% CI, 0.56–0.999) compared to those who reported less constipation severity. Confidence to manage symptoms and excellent adherence were positively correlated at timepoint 3.

High confidence to seek care for symptoms was estimated by symptom severity in separate logistic regressions for each symptom, at all timepoints, but results were not statistically significant (S.I. Table 4) While confidence to seek care was positively correlated with excellent adherence at timepoint 1, the lack of significant correlation with any symptom led us to focus only on estimating confidence to manage symptoms in our mixed effects modeling.

Excellent OAA adherence was estimated by symptom severity in separate logistic regressions for each symptom, at all timepoints (S.I. Table 5). At timepoint 1, participants with more severe nausea had lower odds of excellent adherence (OR = 0.68; 95% CI, 0.49–0.93); each one-point increase in nausea severity led to a 32% lower odds of reporting excellent adherence than participants with less severe nausea. No symptoms were statistically significant in estimating excellent adherence at timepoint 2. At timepoint 3, those with greater tingling/numbness severity had lower odds (OR = 0.66; 95% CI, 0.48–0.88) of reporting excellent adherence than participants with less severe tingling/numbness.

### Evaluating confidence while accounting for time effects

In separate mixed effects logistic models inclusive of all timepoints to estimate factors associated with high confidence to manage OAA, higher severity of the following symptoms was significantly associated with decreased odds of high confidence to manage symptoms of depression, tiredness, drowsiness, constipation, and tingling/numbness (Table 3). For example, participants with greater depression severity had 20% lower odds of reporting high confidence compared to those with less severe depression (OR = 0.80; 95% CI, 0.65–0.97). Participants with greater tingling/numbness severity had the lowest odds of having high confidence to manage symptoms—a one-point increase in tingling/numbness severity was associated with a 23% decrease in odds of reporting high confidence compared to those with less severe tingling/numbness (OR = 0.77; 95% CI, 0.60–0.94). Older age, each additional year, was associated with 4% increased odds of high confidence to manage OAA (OR = 1.04; 95% CI, 1.01–1.08) than younger age. In every mixed effects logistic model, participants were more likely to have greater confidence to manage symptoms at timepoint 2 versus timepoint 1 (Table 3).

An insufficient number of participants with OAA nonadherence precluded the use of mixed effects logistic regression to model OAA adherence as an outcome.

## Discussion

Most participants reported high OAA confidence to manage symptoms and excellent OAA adherence during the first eight weeks of OAA treatment in this exploratory, single-center cohort study. Despite relatively high confidence and adherence, participants reported severe symptoms at all three timepoints. Some lower confidence to manage symptoms and nonadherence remained at the end of eight weeks, with 26% of participants rating confidence to manage symptoms as ≤ 6 and 20% reporting nonadherence (less than excellent) at timepoint 3. These nonadherence rates are within the range reported (0–54%) in existing literature [29–32].

Targeted OAA self-administration increases patients’ symptom monitoring and managing burden while decreasing clinician oversight. We had anticipated many reports of severe symptoms and their persistence throughout treatment. Our findings demonstrate symptom severity and type fluctuate over time. These findings are clinically important, making a case for close continual symptom surveillance, assessment, and support for patients on targeted OAAs—especially during the first several weeks after initiation. These are among the first results to follow patients taking targeted OAAs over time and assess symptomatology alongside confidence to manage symptoms and OAA adherence. Previous studies have similarly found high symptom burden in patients taking OAAs, with considerable numbers reporting severe symptoms [29, 33].

Confidence and self-efficacy increase patient activation, which motivates patients to perform positive health behaviors like symptom self-management [11–13, 17, 25]. Successful symptom self-management then helps increase self-efficacy to self-manage symptoms, as do positive symptom experiences like reduced severity and positive outcomes like enhanced quality of life. These concepts influence each other and are also affected by patient characteristics (Fig. 1) [11–13, 17, 25]. In this study, we used confidence and self-efficacy interchangeably to refer to patients’ belief in their ability to manage symptoms. Our conceptual model considers self-efficacy to be behavior, not symptom, specific [11–13, 17]. Our study participants had better confidence than expected, however, uncovering low confidence 8 weeks into treatment for some participants is concerning clinically, potentially indicating presence of severe unmanageable symptoms and/or inadequate support. Our observed connections between confidence to manage symptoms and symptom severity were also observed in patients with lung cancer, whose increasing symptom severity was associated with lower self-efficacy to manage symptoms [34]. Experiencing severe symptoms decreases quality of life and increases the potential for OAA nonadherence [4, 9]. Self-efficacy promoting interventions in adult patients with cancer and other conditions can help decrease symptom severity and increase quality of life [12, 35]. Successful interventions have included nurse-led motivational interviewing, health coaching, patient diaries for symptom communication and medication scheduling, and nurse-led symptom management education tailored to patients’ physiologic and psychological states [12]. In patients taking OAAs, those with higher confidence and greater patient activation have had better adherence and were more effectively managed symptoms [13, 36].

When estimating confidence to manage symptoms, participants were less confident with more severe tiredness and anxiety at timepoint 1, drowsiness and tingling/numbness at timepoint 2, and constipation at timepoint 3. Participants reporting excellent adherence were more likely to report higher confidence to manage their symptoms at timepoint 3. Our findings indicate efforts to increase patient confidence are warranted and align with previous studies that found patients with higher confidence and activation were able to manage symptoms more effectively [13, 36]. Patient self-efficacy is an important, actionable mediator for self-care behaviors like symptom self-management [37]. Greater symptom severity negatively impacts patient confidence to manage symptoms and, since patient confidence is a mediator for performing self-care behaviors like symptom self-management, inadequate confidence may lead to unmanaged and worsening symptoms [37]. This negative feedback loop worsens quality of life and negatively affects OAA adherence. Multiple studies have found statistically significant associations between patient nonadherence and OAA side effects/symptoms [38–40]. OAA nonadherence is also related to patients’ avoidance of side effects [41]. Our study participants’ high adherence rate could be attributed to the large proportion with metastatic cancer, since patients with metastases prioritize taking OAAs more than those without [42].

Our findings of symptom severity associated with decreased odds of excellent adherence aligned with others who found patients with greater symptom severity had lower OAA adherence [29]. This may indicate poorly managed bothersome symptoms and/or low confidence to manage symptoms contribute to OAA nonadherence, further highlighting the importance of appropriate symptom management and potential for enhanced clinical support.

Numerous studies have shown PROMs enhance standard cancer care [43, 44]. Measures assessing adherence and confidence to manage symptoms could highlight problems for clinicians to target by flagging patients needing more support. Future research should consider developing a composite score for symptom severity and confidence that can gauge patients’ OAA tolerance and identify needs for more support. In patients with advanced malignant pleural mesothelioma, a composite score allows clinicians to quickly evaluate patients for symptom burden, quality of life, and disease worsening [45]. A valid and reliable composite score created for targeted OAAs may enhance care while minimizing clinician assessment and patient completion times.

Study limitations include the small sample size, mostly White participants, observational design, and single center recruitment from a large academic NCI-designated cancer center. The small sample size and homogenous demographics also limit the generalizability of our findings, which are exploratory and should be investigated further in confirmatory studies. Study participants’ direct access to and awareness of extra support at our study site may have inflated their confidence levels, underestimating a typical patient’s needs. Extra site support includes a pharmacy team dedicated to all cancer center patients starting OAAs staffed with pharmacists educating patients and pharmacy technicians providing logistical assistance (e.g., insurance prior authorizations, payment, and drug procurement).

## Conclusion

Many participants experienced severe symptoms during their first 8 weeks of OAA therapy, but the majority reported excellent OAA adherence. Symptom severity peaked within the first few weeks following OAA initiation, indicating the need for early intervention. Participants’ confidence to manage symptoms increased the longer they remained on OAA therapy. Confidence to manage symptoms was inversely related to the severity of depression, tiredness, drowsiness, constipation, and tingling/numbness. These exploratory findings suggest that patient confidence to manage symptoms should be further evaluated, alongside OAA adherence, for viability as a target for clinical interventions aiming to identify patients needing more support and/or enhanced symptom management while taking OAAs.

## Data Availability

Data are available upon reasonable request to the corresponding author.
